# “As a Parent You Become a Tiger”: Parents Talking about Bullying at School

**DOI:** 10.1007/s10826-017-0710-z

**Published:** 2017-04-11

**Authors:** Rebecca Hale, Claire L. Fox, Michael Murray

**Affiliations:** 1 UCL Institute of Education, 20 Bedford Way, London, WC1H 0AL UK; 2 School of Psychology, Keele University, Staffordshire, UK

**Keywords:** Parent, School, Bullying, Peer victimisation, Teachers

## Abstract

Bullying at school can be a distressing experience for children. It is also likely to be distressing for their parents. In spite of this, research in the field of school bullying and peer victimisation has tended to overlook the experience of parents when their child is bullied. This study explored school bullying from the parent’s perspective. Twenty-one parents took part in semi-structured focus groups and interviews to share their experiences. Thematic analysis of the interview transcripts identified two main themes: ‘perceived institutional factors’ and ‘being a good parent’. It was found that parents viewed their principal role as protecting their child; they referred to this as an instinct and fundamental to them being a good parent. However, during their attempts to help their child, many parents talked about difficulties working with schools and this triggered frustration and distrust towards teachers. The findings highlight the importance of communication between parents and teachers and ensuring that parents are kept informed of progress when teachers are trying to address the problem. Additionally, the findings indicate that parents may hold different views to teachers about their role in school bullying situations. This would suggest that parents looking at the situation from the teacher’s perspective, and vice versa, could help to build better parent–teacher relationships when tackling school bullying.

## Introduction

In bullying research, the lived experiences of parents have typically been overlooked. Parents have been referred to as ‘the missing voice’ in bullying literature (Sawyer et al. 2011), as well as ‘secondary victims’ (Sullivan et al. 2004). A review of studies using qualitative methodology demonstrated that only a limited number had examined parents’ perspectives on school bullying (Harcourt et al. 2014). Consequently, little is known about how parents feel when their child is bullied, how they react, and their experiences of helping their child.

Prevalence rates for bullying vary across studies, but a recent review of literature identified that between 10 and 33% of students reported being the victims of bullying (Hymel and Swearer 2015). Bullying involves repeated aggression towards someone who cannot defend themselves, with the intention of causing them harm (Olweus 2013). Behaviours can include physical, verbal, relational and electronic forms, and be further subdivided into covert and overt actions. These forms of bullying can be experienced simultaneously, and research has indicated that verbal bullying often accompanies relational and/or physical forms (Bradshaw et al. 2015). Indeed, it is important to recognise the complex patterns of behaviour that characterise bullying incidences. For example, Ging and O’Higgins Norman (2016) found that online victimisation amongst girls were often reflective of ongoing and public tensions in the school environment.

The form of bullying experienced by children is likely to have an impact on their parents. For example, evidence has suggested that some parents viewed different forms of bullying as more serious than others and were more likely to respond to their child’s victimisation if it escalated into physical forms (Harcourt et al. 2015; Sawyer et al. 2011). Parents may also be unaware that their child is being bullied. A recent study by O’Higgins Norman and McGuire (2016) found that 16.5% of parents never or rarely spoke to their children about cyberbullying and Tokunaga (2010) noted that young people were reluctant to tell their parents about online victimisation because of fear that their access to the Internet would be removed.

Where research has looked at parents’ emotional responses, findings have revealed how distressing this experience is for them. Parents have reported their ongoing distress in tandem with their child’s continued victimisation (Rigby 2008), their own anger and their concern about the impact of the bullying on their child (Sawyer et al. 2011). A study by Brown et al. (2013) found that parents felt helpless when their child was bullied, especially when they thought the school was not responding in a way that would protect their child or prevent the problem from reoccurring. Other studies have also reported parental frustration at the limited response of the school when their child is bullied. Holt et al. (2009) found that 81% of parents thought that schools should be more responsive to bullying problems, Greeff and Van den Berg (2013) found that parents were often dissatisfied with the school’s response and Harcourt et al. (2015) reported that parents typically did not experience positive or active responses from their child’s school.

Research indicates a number of reasons why parents do not always think schools do enough. Atlas and Pepler (1998) found that teachers did not intervene in bullying incidents because they had not seen them occur. This was because students often engaged in bullying when the teacher was not looking. Research has also suggested that teachers are reluctant to respond to reports of school bullying unless they can be confident of what really happened, for example by witnessing it themselves (Hein 2016). Additionally, evidence suggests that complaints of physical and verbal bullying are likely to be taken more seriously than relational bullying because teachers do not always view social exclusion as a form of bullying behaviour (Naylor et al. 2006; Yoon et al. 2016). This could influence the extent that teachers intervene in more covert types of bullying.

Studies have also indicated that schools differed in the implementation (and thus, effectiveness) of anti-bullying policies (Smith et al. 2008, 2012). A content analysis of anti-bullying policies by Smith et al. (2008) found that only half of participating secondary schools had policies that discussed when or how parents would be informed of bullying. A follow-up study 6 years later suggested an improving picture with 91.7% of secondary school anti-bullying policies referring to this action (Smith et al. 2012). However, they found little change in the proportion of policies which included advice for parents about bullying, with 51.9% of secondary schools in the 2008 study doing this compared to 52.1% in the 2012 study. Evidence has also suggested a lack of congruence between the content of anti-bullying policies and the actual actions taken by the school, including statements in student handbooks that said parents would be notified during episodes of school bullying, though this often did not occur (Brown et al. 2013).

Even if schools take parental complaints seriously, parents can encounter a number of difficulties that influence partnerships with the school when tackling the bullying problems. Parents have reported problems in contacting teachers (or head teachers) directly, instead having to report their complaint to the school secretary (Brown et al. 2013). In these circumstances, it can be unclear if messages have been passed on to relevant school staff. Indeed, lack of communication from the school has emerged as a significant issue in parents’ experiences when supporting their bullied child. For example, requests to teachers for more transparency and regular updates about their child’s wellbeing following a bullying incident have been met with reluctance (Hein 2014). Research has indicated that it is more usual for the parent to contact the school for information rather than the other way around (Harcourt et al. 2014).

Another concern is the formality of schools (especially secondary schools) and the position of power that teachers can adopt during interactions with parents. Observations of parents’ consultation evenings showed that teachers gave an uninterrupted ‘diagnosis’ about the child’s progress, achievements and behaviour. If the parent contradicted the teacher’s account of their child’s progress, a process referred to by the authors as “extended blaming negotiations” sometimes took place (MacLure and Walker 2000). Recently, Pillet-Shore (2015) reported similar findings after observing 41 parent–teacher consultations. The study found that the parents endeavoured to present themselves as ‘good parents’ during these interactions by asserting their prior knowledge of their child and explaining their efforts to address any issues the teacher raised. Pillet-Shore argued that parents took this approach to highlight their child’s problems were not due to parental negligence.

As Crozier and Reay (2005) point out, interactions of this nature are likely to foster distrust in the parent–teacher relationship because teachers seek to maintain authority and can feel that their professional expertise is being questioned; whereas parents become frustrated that their expertise as the parent of the child is being overlooked by the teacher. Indeed, studies have shown that parents who questioned teachers’ approaches were viewed negatively by teachers, who became frustrated with the parents and avoided interacting with them (Graue and Brown 2003; Ribbens McCarthy and Kirkpatrick 2005; Walker and MacLure 2005). Moreover, teachers’ descriptions of ‘ideal’ and ‘good’ parents referred to those who refrained from challenging a teacher’s professional expertise and instead supported the work of the school (Bæck 2010; Graue and Brown 2003).

Studies have shown that a common parental response to their child’s peer victimisation is to contact teachers (Greeff and Van den Berg 2013; Harcourt et al. 2015; Holt et al. 2009; Sawyer et al. 2011). Consequently, it is likely that these issues play out in school bullying situations. That is, the teacher takes the lead in how to address the problem and where a parent is perceived to be intervening too often, there is a risk that the teacher will seek to reduce contact with the parent. Recent research has suggested that this may be the case. Hein (2014) found that parents initially endeavoured to work with the school and followed their direction in tackling bullying problems. However, they became increasingly frustrated with the school when their child continued to be bullied. During this process they were keenly aware that they might be regarded by the school as ‘troublemakers’ for doubting the school’s approach and signalling this doubt by instigating ongoing interactions with the school. Although Hein’s study focused on the experiences of a small number of parents, it does provide some insight into the concerns of parents in relation to how they are perceived by the teachers. Harcourt et al. (2015) also found that parents of bullied children were worried that teachers would label them as ‘complainers’ and/or perceive they were trying to dictate to teachers how they should do their job.

The situation outlined is likely to be further exacerbated because parents and teachers are approaching it from different perspectives. Roffey (2004) found that parents believed the role of parents was to protect their child and believed the role of teachers was to also do this by acting *in loco parentis*. This expectation influenced the nature of parent–teacher interactions such that teachers who tried to understand a child were perceived far more favourably than teachers who viewed the child negatively. Addi-Raccah and Arviv-Elyashiv (2008) found that teachers considered their role to be an educator imparting knowledge to students, whereas parents viewed a teacher’s role as caring for their child and acting like a mother.

These considerations are likely to be significant when school bullying occurs because the victimised child’s safety is threatened and the parent will strive to protect their child (Brown et al. 2013; Hein 2014, Harcourt et al. 2015). However, this can be challenging for parents when working with schools to tackle the problem. Roffey’s research highlights the importance of not only examining parents’ experiences when their children are bullied, but also considering how these experiences are subsumed by their broader beliefs about the role of parents (and the role of teachers). Hein’s (2014) research also points to the importance of doing this. Hein found that parents reflected on their actions, doubted their own judgment when their child was bullied, and looked inwards at themselves as parents and what they could have done differently to have prevented this from happening to their child. Thus, it appears that an important element of the parent’s experience when their child is bullied is to reflect on what *kind* of parent they will be perceived to be (Herne 2016; Pillet-Shore 2015).

Parents’ perceptions of the parenting role, especially in situations where their children have been experiencing problems, have highlighted a ubiquitous discourse that is prevalent in society: the good parent and the bad parent. For example, a ‘good’ parent feeds their child the ‘right’ food (Perrier 2010), consults with contemporary parenting research (Pedersen 2016), uses effective discipline strategies without resorting to authoritarian measures (Perrier 2010), and cultivates opportunities for their child to engage in extra-curricular activities (Vincent and Maxwell 2016). Thus, expectations of parents are both abundant and diverse. However, parents will often find themselves confronted with unanticipated and complex problems which hinder their efforts to perform this role and trigger concerns that they are a ‘bad’ parent.

Representations of the parental role are embedded in society and have given rise to judgements that parenting is something that parents either get right or wrong (Gillies 2010), and that professionals can monitor this process and intervene if necessary (Phoenix and Woollett 1991). This has contributed to the dichotomised discourse whereby parents are viewed as either ‘good’ parents or ‘bad’ parents (Reay 2010). This has also impacted on how parents perceive themselves. For example, Henderson et al. (2016) found that mothers who experienced pressure to be perfect expressed guilt for not meeting parenting expectations and reported stress, anxiety, and a low self-efficacy. Research in the UK has also suggested that episodes of media attention on ‘good’ parenting (e.g. a television programme on this topic) can trigger parental self-scrutiny (Pedersen 2016). Consequently, the parental perspective in school bullying situations will be embedded in the broader context of what it means to be a parent.

In summary, research has suggested that when parents approach the school/teachers about bullying they can encounter some difficulties including teachers not always taking parental complaints seriously, the formality of interactions with teachers, and approaching school bullying from a different viewpoint to the teachers. It is likely that a parent’s principal concern is to protect their child. However, research to specifically explore parents’ experiences when their child has been bullied, including how they felt, the nature of interactions with the school, and their views on their own role in tackling the problem have been lacking. The aim of this research was to conduct an exploratory study to find out more about parents’ experiences and gain insight into school bullying from their perspective. The data collected in this study will be interpreted within the theoretical framework of parents’ perceptions of their parenting role.

## Method

### Participants

Secondary schools and community centres (which had parenting groups) in the North West of England were approached by letter to explain the research and request permission to contact parents about the study. The study was also advertised to staff and students at the university where this research was conducted. Where schools and parenting groups expressed an interest in participating, the first author of this paper liaised with a head teacher, teacher or group leader as appropriate. These individuals identified parents whose children had experience of being bullied. A letter from the researchers was sent to the parents to invite them to participate.

The breakdown of participants is given in Table 1 (the parents’ names have been changed to protect their identity). Where the participants were recruited at the university, the first author communicated with them directly throughout the process. In the case of schools and parenting groups, staff at these institutions made initial contact with the parents. It is acknowledged that because of this there would have been some bias in the parents that were approached. For example, a head teacher might be more likely to identify parents who they think will give a favourable account of how the school tackled the problem. However, as the findings will show, the parents disclosed negative experiences and views of the school, and felt able to do this because they were assured of their anonymity and that the school would not have access to the recordings or transcripts of the interviews and focus groups. The process of recruiting parents to this study proved challenging which was demonstrated by the different settings in which parents were contacted and the reliance on schools to help with recruitment. While this may have contributed to a biased sample, it was important to utilise the assistance of teachers to both identify relevant parents and to provide initial communication channels between the parents and the researcher.

**Table 1:** Overview of participants

Focus group/interview	Location/recruitment method	Name of parent	Bullied child(ren)
Focus group 1	Community centre, parenting group. The group leader invited parents who she knew had experiences of their child being bullied	Lucy	Female, adolescent; Male, adolescent
		Olivia	Female, adolescent
		Ruby	Female, adolescent
Focus group 2	Community centre, parenting group. The group leader invited parents who she knew had experiences of their child being bullied	Anna	Female, adolescent
		Dee	Male, adolescent
		Ellie	Male, pre-school
		Hollie	Male, child
		Sarah	Male, adolescent
Focus group 3	School 1. Non-teaching staff at the school (e.g. lunchtime assistants) were sent an email by the head teacher and asked to contact the researcher if they were interested in participating	Amber	Female, child
		Shelly	Female, adolescent
		Zoe	Female, adolescent
Paired interview 1	Keele University. Using university mailing lists, an email was sent to staff and students at the university	Heidi	Female, adolescent
		Miriam	Female, adolescent
Paired interview 2	School 2. Teaching staff at the school identified and invited the parents to participate	Honor	Male, adolescent
		Kendra	Male, adolescent
Individual interview 1	School 3. Teaching staff at the school identified and invited Amara to participate	Amara	Male, adolescent
Individual interview 2	School 3. Teaching staff at the school identified and invited Diane to participate	Diane	Male, adolescent; Female, adolescent
Individual interview 3	Keele University. Using university mailing lists, an email was sent to staff and students at the university	Lily	Female, child
Individual interview 4	Keele University. Using university mailing lists, an email was sent to staff and students at the university	Lorraine	Male, adolescent
Individual interview 5	School 3. Teaching staff at the school identified and invited Martha to participate	Martha	Male, adolescent
Individual interview 6	Keele University. Using university mailing lists, an email was sent to staff and students at the university	Phoebe	Male, adolescent; Female, adolescent

Overall, three focus groups and eight interviews (six individual and two paired) were conducted giving a total study sample of 21 (female) parents/carers. Sample sizes in qualitative research are often guided by the concept of data saturation. Saturation is thought to have occurred when analysis does not reveal any new categories in relation to the central issue being researched (Chamberlain 1999). This approach was applied in this study. Thus, as data were collected a provisional theory that accounted for the data was developed. Subsequent cases were reviewed in relation to this and adjustments were made to the themes as necessary. Once reviews of additional data from more participants did not change the themes, recruitment stopped.

Two of the focus groups took place at the community centre and one took place at a secondary school (School 1). Four interviews took place at the university, one took place in School 2 and the other three interviews took place in School 3. Where interviews/focus groups took place at the university and in the community centre, the parents were making reference to different schools when they talked about their children’s experiences (that is, none of the children discussed attended the same school). Age group given is the age of the child when parents participated in the study, ‘pre-school’ refers to children aged up to 5 years, ‘child’ refers to primary school age 5–11 years, and ‘adolescent’ refers to secondary school age 12–16 years. As indicated in Table 1, across the sample of 21 parents the experiences of 24 young people were referred to (three parents talked about two of their children being bullied). There was an even split of boys and girls, and the majority of the young people were adolescents (*n* = 20), with three children in primary school and one child who was in pre-school. The higher proportion of parents of adolescents occurred because secondary schools were contacted during the recruitment phase as evidence has suggested there is a peak in the prevalence of young people being a victim of bullying in England when in the lower secondary school years (Eslea and Rees 2001). However, some parents of younger children who were aware of the study (for example, through the parenting group) expressed a wish to participate, and so they were also included.

### Procedure

A qualitative design was used involving a combination of focus groups and interviews. Focus groups were used because they provide a setting that can support and encourage participants to share their views and help them develop their viewpoint through discussion with others (Vaughn et al. 2000; Sagoe 2012). However, a drawback of focus group research is that some people may be reluctant to share their experiences in a group setting (Krueger and Casey 2015). Thus, interviews were conducted with parents who preferred this approach.

Ethical approval for the research was gained from the university research ethics committee and followed the British Psychological Society’s Code of Ethics and Conduct (2009). The focus groups and interviews took place in schools, community centre parenting groups and at the university. Parents were assured that the full discussions would not be shared with anyone (including teachers), and only short anonymous quotes would be used in research reports (with their permission). Parents were asked to only discuss experiences they felt comfortable to reveal, and those participating in focus groups/paired interviews were asked not to disclose the content of the discussions with any individuals outside the group. Informed consent was obtained from all participants. Interviews lasted approximately 30 min; focus groups ranged from 30–60 min. A schedule of questions was used to help guide the discussion, but a semi-structured format was used so that parents could discuss anything they thought was relevant. The questions started by asking parents to talk about definitions of bullying and what they thought caused it. Parents were invited to talk about their own experiences of their child being bullied including how they found out, how they reacted, the outcome of their child’s experience, and their viewpoints on how schools could handle bullying situations. These questions were presented in a neutral and open format, for example asking parents to talk about their experiences without stating they should talk specifically about negative and/or positive episodes. The discussions were audio recorded.

### Data Analyses

The focus groups and interviews were transcribed by the first author of this paper. During this process initial thoughts were noted down and the analysis sought to derive ideas and themes that emerged from across the interview/focus group and not just from a single question. The transcripts were first coded within NVivo. Each transcript was read closely and coded sentence by sentence; nodes were created to represent their content. For example, one parent said “as far as the school is concerned, lip service to an anti-bullying policy I think” and this was initially coded in an *anti-bullying policy* node. Another parent said “I kept thinking I must have done something wrong” and this was coded in a *parental self-blame* node.

NVivo was used in the early stages of coding as a tool for organising and managing the data. Analysis then progressed to higher level interpretation, including the merging of codes into themes. This was done manually by printing out each code (node) and corresponding text segments. The text linked to each code was reviewed and candidate themes were identified through considering the relationships between codes. This process was guided by the analysis steps outlined by Attride-Stirling (2001) and Braun and Clarke (2006). In many instances recurring patterns in parents’ accounts were found and these accounts were often in relation to parents’ negative experiences. Where divergent views or atypical experiences were expressed, they were included in the relevant themes. For instance, occasionally parents made reference to positive experiences or outcomes when helping their bullied child (typically in situations where their child had moved to a different school or a new head teacher had started at their child’s current school) and these are included where relevant as a contrast to the more prevalent parental experiences. All three authors discussed at length the identification and refinement of the sub-themes and themes, interpretations of the data, and how the themes related to existing literature in the field.

This process identified a number of themes, which were reviewed in light of the data extracts and the whole data set. Subsequently, some themes were refined by merging them with other overlapping themes. Other themes were modified to account for the data extracts they denoted and ensure the themes were a true representation of these data. This process facilitated the identification of several sub-themes: school policies; perceived school views of parents; parent–teacher communication; protecting their child; and appraising self and others. There were relationships between the sub-themes, and consequently the arrangement of the themes was considered to explore how they appeared to group together to signal overarching themes. This element of the analysis led to the identification of two main themes: perceived institutional factors and being a good parent. Each of these main themes and the corresponding sub-themes are discussed with relevant extracts from the focus groups/interviews.

## Results

### Perceived Institutional Factors

Parents’ encounters with the school were often numerous and varied, typically commencing with a telephone call to the school to make a complaint about bullying, followed by a series of face to face meetings and/or further telephone conversations with teachers. Parents believed the role of the school was to discipline the pupils when they misbehaved (as in the case of pupils who bully), protect children (especially in circumstances where their child’s safety was at risk, such as bullying) and communicate with the parents (something parents often felt was overlooked when the school was tackling the victimisation experiences of their child). The majority of parents reported that the teachers did not communicate with them and appeared to do very little, thus putting their child at further risk by allowing the bullying to continue. These concerns meant that parents often felt unable to control the situation and perceived that barriers existed between themselves and the school, contributing to a sense of distrust.

#### School policies

Some parents did not believe teachers when they said they did not know about the bullying problem and suspected that the teacher had not attempted to tackle the problem because of other priorities. These priorities included adhering to the anti-bullying policy procedures (even if the parent thought they were ineffective) and only intervening when the bullying became more serious. Consequently, these motives were then perceived by parents to increase the risk to their child because the bullying was not being addressed, which created a sense of distrust towards the school.

“Seemingly no teachers or playground staff had known that this was going on.”

(Phoebe, individual interview 6)

“I found it a bit odd because it was, a lot of [bullying] was on the playground or on the bus, you know, and I’m thinking well somebody must have known, you know and it turned out there was another girl that they were doing it to as well, erm but how they could say they didn’t know?”

(Miriam, paired interview 1)

Overall, there appeared to be a lack of confidence in their child’s school’s anti-bullying policy which showed that in general these policies were not valued by parents. It should be noted that because the parents were referring to experiences at different schools, the content of the policies they referred to also varied. This issue was not examined in this research (for example, to ascertain the extent that the relevant schools did or did not adhere to their policies), however, many of the parents in the focus groups/interviews held negative opinions towards the policies, for example:

“I didn’t get any joy from the [anti-bullying policy] to be honest. I heard about it and apparently they’ve got a policy at the school, they’ve got a policy but it’s, it’s almost like they try to ignore it.”

(Miriam, paired interview 1)

Miriam’s comment suggested that not only did she not value the anti-bullying policy, but she also doubted that the school valued the policy. Other parents shared her sentiments, tapping into the idea that the anti-bullying policy had two aspects. One aspect was the anti-bullying policy as a document which outlined what the school would do in an incident of bullying, and the other aspect referred to the actual actions taken by the school when bullying occurred. However, parents identified that there was no correspondence between these two aspects and the anti-bullying policy was a source of contradictions in the school’s handling of bullying situations:

“I feel as though erm it doesn’t matter how good the policy is if they’re not implementing it, it’s not going to do anything.”

(Olivia, focus group 1)

“You can’t just have a policy and stick it on a wall somewhere without making sure that that policy is embedded into the culture of the school, and you know, in most schools you don’t hear of them tackling bullying head on.”

(Heidi, paired interview 1)

Three parents, Honor, Kendra and Phoebe, praised the anti-bullying policies that were in place at the schools that their children currently attended. For example:

“I think [the school] is doing a lot of the right things now, so they do the right things, and an incident happens, they question the people involved separately, they question the witnesses, so they find out what went on, so the school have got a clear picture of what went on, and they inform the parents on both sides as to what’s happened, and give them as clear a picture as they can about it.”

(Phoebe, individual interview 6)

Phoebe’s view highlighted the importance of a direct and consistent approach from the school, where parents were kept informed about what was happening and felt confident in the school to “do the right things”. However, the three parents who praised current anti-bullying policies were also able to cite previous bad experiences (either at primary school or their child’s current secondary school when it was under the leadership of a different head teacher) in relation to the ineffective implementation of anti-bullying policies. This discontentment towards the anti-bullying policy contributed to the distrust towards the school because the parents felt the policies were ineffective. They existed because they legally had to, and in a lot of cases the school had referred the parents to the policies as evidence that the school had procedures in place to tackle the bullying. In this way, rather than reassuring parents, anti-bullying policies were perceived by parents as more of a barrier and something that schools ‘hid’ behind.

#### Perceived school views of parents

A source of frustration in parents’ experiences was that they felt the school did not always believe them when they reported their child’s victimisation and/or the school did not think the incident was as serious as the parent perceived it to be. One parent, Miriam, reported that her concerns were not taken seriously and she thought this was because her daughter was bullied verbally and through social exclusion, rather than being physically bullied.

“[The school] didn’t seem to take it seriously. I think, like I say, if somebody had punched a child, or threatened to knife them, I know they’ve had trouble with drugs which they do everywhere, that would have been a big deal. But to me it was a big deal: that’s my daughter.”

(Miriam, paired interview 1)

Some parents engaged in a process of collecting ‘evidence’ to demonstrate that their child had been bullied in case the school doubted their complaint. This evidence included physical marks on the child like cuts and bruises, witnesses who saw the bullying take place, computer screen shots and text messages to demonstrate cyber-bullying and damage to the child’s property. This gathering and presentation of evidence to the school appeared to serve two purposes: to justify approaching the school in the first place and to support the seriousness of the complaint the parent was making.

“There was quite a lot of text messages that I’d said to my daughter don’t delete it, and she had deleted it, and you know you mustn’t delete anything like that that you get, you mustn’t delete it because that is your evidence that it’s going on.”

(Zoe, focus group 3)

“I kept phoning school up and said “look I want to bring him back [to school]” but there was nobody, they couldn’t find my son’s head of year to show the things. In fact I brought my son back the next day and said “look, this is what happened”, and my son had a bump on his head, scratches.”

(Amara, individual interview 1)

“He went to see the head of year and his friend went with him and said what had been happening with [the peer victimisation] as almost like a witness really.”

(Lorraine, individual interview 4)

Parents’ perceptions that the school did not believe them appeared to reflect an underlying concern that the school did not value their contributions during the process of dealing with their child’s victimisation problems. Parents wanted to be involved in issues related to their child’s well-being, and they wanted the opportunity to help. They endeavoured to protect their child from the bullying, but struggled to do this. This meant they often felt excluded from the process.

“Everything has got to be on [the teachers’] terms, and I mean I don’t know about you, but you feel inadequate enough already.”

(Miriam, paired interview 1)

“I think parents, you’re out on a limb really because the school environment, you’re an outsider, you’re not part of that school. As much as they say that they want you part of that school life, you’re not here. The school life is for the teachers, people that work here and the children. We’re sort of on the peripheries.”

(Honor, paired interview 2)

“I think some, not all obviously, but some teachers haven’t got time to listen and I think that’s a problem as well isn’t it?”

(Zoe, focus group 3)

In contrast to these experiences, three parents (Honor, Kendra and Phoebe) found that their child’s current school did believe their complaints and responded quickly. Consequently, these parents expressed positive opinions about the school, and had confidence in the staff to effectively tackle bullying incidents:

“I dropped my son off the next morning and the learning support teacher came running across the car park and said “I sorted it for you; the boy has admitted it and he will be punished for his actions”. I was happy with that, my son was happy with that, so I was extremely happy.”

(Honor, paired interview 2)

#### Parent-teacher communication

Experiences of parent–teacher communication were frequently discussed by parents. For some parents, school procedures made it physically difficult for them to have any form of communication with the teacher (for example, having to go through the school receptionist to talk to a teacher on the telephone). However, for most parents in this study the main communication issue occurred because they reported that the teachers did not keep them informed about bullying incidents that had happened to their child and actions taken to tackle the bullying.

“I don’t think they get back to the parents the way that they should and get back to the child and say “is everything okay?”.”

(Olivia, focus group 1)

“Well I tried to see the headmaster and you had to make an appointment like 3 weeks in advance, and you know by the time you got to see him, he was like “oh well it didn’t happen”.”

(Anna, focus group 2)

In contrast, the three parents who were satisfied with the response of their child’s current school and/or current head teacher talked about the school’s consistent contact:

“It’s a lot better now, and now when I get onto the school about anything you will get a phone call or an email the same day normally, and if not, it’s going to be a day later, and if it’s something important they, they will.”

(Phoebe, individual interview 6)

When schools failed to contact the parents and/or institutional factors prevented parents from contacting a specific teacher, parents were left to decipher for themselves why the bullying had occurred and how the school was dealing with the problem. In many instances, teachers may well have acted to address the bullying incidences that were reported. However, parents were not made aware of this and so when their child continued to be bullied they took this as an indication that the school had not responded, for example:

Amara: I think when they actually go in and say, you know “my son is being bullied” (..) sometimes it doesn’t seem like [the teachers] are actually doing anything, do you know what I mean? It don’t feel like they’re actually wanting to do much until it comes to like the crisis point.

Researcher: are they telling you that they’re doing something? ﻿Amara: sometimes they’ll say “oh yes, we’ve had a word with them, we’ve had them in the office and had a word” but it don’t seem like it.

Researcher: do you get the feeling/

Amara: the feeling that it doesn’t seem like they’re actually doing anything.

Researcher: why do you feel like that? Amara: because it’s still happening.

(Individual interview 1)

#### Summary

All of the parents contacted the school believing the teacher would address the peer victimisation problem. However, parents’ accounts indicated they often had negative experiences when working with schools in these circumstances. Their comments pointed towards distrust in schools, and this situation was exacerbated by their inability to control what happened to their child at school. This sense of helplessness was further exacerbated when teachers did not keep in contact with the parent and/or referred them to anti-bullying policies, without (as the parent perceived it) actually following any of the guidelines they contained.

It should be noted that the parents’ accounts were in reference to children across a broad age range, and it is acknowledged that stage of schooling will have had an influence on parents’ experiences. In terms of perceived institutional factors, parents noted that at primary school there were regular opportunities to speak face to face with teachers, for example when taking their children to and from school. In contrast, they felt more on the peripheries at secondary school and as Honor explained “even though [secondary schools] say it’s one big family, you’re not, you’re outside, you’re at the gate, whereas you can get inside the gate at a primary”.

### Being a Good Parent

The parents in this study endeavoured to help their children. They saw this as a key part of the parental role; something that good parents did. The concept of the good parent emerged through two different strands. Firstly, parents highlighted their role as the protector of their children, and this was linked to distress at being unable to control the situation (and thus, being unable to protect their child). Secondly, in light of the link they made between protecting one’s child and ‘good’ parenting, discussions sometimes progressed to self-assessment and in turn self-blaming. Accordingly, the parents evaluated their own actions within the parental role.

#### Protecting their child

The focus groups and interviews showed that the aim of the parent, in whatever action they took to tackle the bullying, was to protect their child. This was seen as one of the most fundamental roles of being a parent, especially in the case of bullying where their child’s safety was at particular risk. It was talked about as being instinctual and appeared to be a key responsibility in being a good parent:

“Your automatic reaction is to protect your own kid.”

(Zoe, focus group 3)

“As a parent you become a tiger where your children are concerned and you can shout at them and tell them off, but nobody else does it, and somebody does something to upset your child to that extent, then it’s both barrels blazing, you come in and you’re ready to take the world on.”

(Honor, paired interview 2)

Some parents became so frustrated and angry by the situation that they developed feelings of aggression and resentment towards the bully. This anger was evident in comments where they referred to wanting to ‘strangle’ the bully or ‘punch their lights out’. This instinctual response was simultaneously accompanied by an acknowledgment that being aggressive towards the child who was bullying their child was not an appropriate response.

“I felt like I wanted to strangle [the bullies] and I know you can’t do that, you know. Because it doesn’t matter how old your child is they’re still your babies.”

(Miriam, paired interview 1)

“You’ve got to be strong for your kids, it’s emotionally draining, it does upset you, you feel like you want to go and punch [the bully’s] lights out.”

(Zoe, focus group 3)

“I feel total absolute white rage. I can feel it now, the rage of panic that my kid’s being devastated every time she goes out and what am I going to do about it? Am I going to kill this kid or this group?”

(Olivia, Focus group 1)

Although parents could protect their child at home, they were unable to protect their child outside the home environment and this was especially the case with situations that occurred within the school. For some parents, school appeared to be a very dangerous place to send their child, especially if they perceived the school did not provide any protection for their child.

“They spit at my son, they take things off him, do you know what I mean? Pushing and prodding him all the time. How can you send your son on a school bus like that?”

(Amara, individual interview 1)

“I got myself so anxious about my son going out to school it seemed the least safe place in the world he could be.”

(Phoebe, individual interview 6)

Parents’ views that the environment outside the home was unsafe sat uncomfortably with their recognition that as their child progressed through adolescence there was less that they could do to protect their child. Consequently, parents faced a paradoxical situation of needing to protect their child, whilst also granting them increasing independence. It should also be noted that this was a particular concern to the parents of secondary school children.

“It was almost a wake-up call to think, hang on, she’s out there in the big world now, you know, in a few years time she might be going to university whatever, and it’s yeah, I’m just aware now that she’s growing up and really at the end of the day there is only so much you can do. I mean you can’t physically lock them indoors as much as you’d like to sometimes.”

(Miriam, paired interview 1)

“I can’t wrap him up in cotton wool, you’ve got to see that you can’t wrap him up, but there is a limit for what you will subject your child to.”

(Amara, individual interview 1)

In most day to day situations parents could control what happened to their child, and in doing so, also help to solve any difficulties their child experienced. However, their child’s victimisation not only represented a situation where they struggled to protect their child, but also a situation where they had little or no control over if and when the bullying occurred, and whether or not the bullying was tackled by the school.

“What I can’t control is what goes on at school. And all this stuff seems to go on at school, or on the bus, so what can I do? If I can’t penetrate the school environment then I’ll just have to let her go and put up with it don’t I? And that’s not acceptable.”

(Heidi, paired interview 1)

“I’d never had any problems before as such, and then when [bullying] happens, you’re so out of control aren’t you? You can’t do anything about it.”

(Dee, focus group 2)

#### Appraising self and others

Parents indicated that their endeavours to protect their child were embedded in a broader context of what it meant to be a good parent. For example, when their child became the victim of bullying, some parents perceived that they were somehow responsible for this situation. This self-evaluation contributed to them experiencing feelings of self-blame, self-doubt and anger towards themselves.

“Why is it happening now? You know, why? What have I done wrong or what haven’t I done? Or what, you know, and in my head I went through the whole thing. Well should we have stayed in London? Should we have done this? What if I hadn’t got divorced? What if?”

(Miriam, paired interview 1)

“I was extremely angry, one with myself for not pursuing the matter and really getting to the bottom because I knew something was wrong because of the way he was behaving. So I was angry at myself.”

(Honor, paired interview 2)

This process of self-evaluation was also indicated in a quote from Anna who, when trying to make sense of her child’s victimisation, deemed herself responsible because she had told her daughter it was wrong to hit. In Anna’s eyes this had made her daughter an easy target.

“I felt guilty because I told her she weren’t allowed to hit people, and if she’d smacked the first one back they wouldn’t have carried on, because they just saw her as an easy target.”

(Anna, focus group 2)

Parents’ self-awareness of their parental role was also shown in their judgements about their responses. Some parents indicated regret for not responding in a particular way to the bullying, or doubted the actions they did take. For example, Heidi was hesitant about the advice she gave to her daughter to end a friendship with a girl who went through phases of socially excluding her daughter and spreading her secrets. Lily had changed her daughter’s school because of the continued victimisation, but was concerned that she should have been more assertive with her daughter’s previous school. Lorraine’s response was to approach the school when her son was physically bullied, but the school’s response made her wish she had contacted the police.

“I was advising her, maybe wrongly, that this friend wasn’t good for her.”

(Heidi, paired interview 1)

“We felt very much alone in this erm we didn’t necessarily think that it was taken that seriously by the school, and admittedly we probably could have kicked up a bit more of a fuss about it.”

(Lily, individual interview 3)

“My biggest regret in all of it is that I didn’t call the police. That was my biggest regret, because that is all the recommendations that the school gave “you should have called the police over this and got the police officers, community support officers involved”.”

(Lorraine, individual interview 4)

Finally, parents’ self-appraisals about their handling of their child’s victimisation were often talked about within the wider context of being a parent. This included their judgements about themselves and other parents in relation to how they raise their children on a day to day basis.

“I think as a parent, I want more than [school policies] you know, because I feel that I brought my children up in a sound environment with a sound outlook and I want the school to talk to me about behaviour as well.”

(Heidi, paired interview 1)

“I do have consequences and they do, you know, they will get sent to their rooms, and they will have their pocket money docked if they do anything badly, and they will be kept in, you know if they did something bad.”

(Phoebe, individual interview 6)

“I think parents have a huge part to play. You don’t like being told, well I can imagine you don’t like being told that your son’s just being caught attacking another child erm cause you know, your child’s an angel in your eyes, you know, no matter what they do, erm but some days you have to learn to deal with that.”

(Lorraine, individual interview 4)

The quotes demonstrate that although these parents often doubted their actions when helping their child to tackle peer victimisation and in some cases felt guilt and self-blame for their child’s experiences, when appraising themselves as parents from a more holistic perspective, they were more positive about themselves. They highlighted examples of how they had been good parents, for example, by punishing bad behaviour. Consequently, they directed frustration towards parents who did not do these things, and so were responsible for their children’s bad behaviour. In Heidi’s case, by fulfilling her role as a good parent, she was frustrated at the teachers for not acknowledging this and in turn adhering to their role in reinforcing the values that she had instilled in her children at home.

#### Summary

For these parents, protecting their child was instinctual and fundamental to the parental role. However, parents appeared to experience some dissonance by recognising themselves as a good parent on a daily basis, but not being able to fulfil the principal task of a good parent (to protect their child). This led to feelings of distress and anger towards the bully, and frustration that they lacked control over the situation. For parents of adolescents, their automatic response to protect their child clashed with their realisation that their child was growing up and would increasingly face challenging situations which they would not always be able to resolve on behalf of their child.

## Discussion

The focus groups and interviews revealed the complex experiences of parents when their children are bullied. All of the parents contacted their child’s school, and evidence has suggested that parents want to work collaboratively with teachers and in a way that does not signal doubt in the teacher and/or leads to the parents being perceived negatively (Harcourt et al. 2015; Hein 2014). The parents in this research explained that they struggled to be good parents and were frustrated by perceived institutional factors as they sought to work with the school to tackle bullying. For example, the parents often did not trust the teachers, they believed they needed to support their complaint of bullying with some form of evidence, they suspected the school already knew about the bullying and crucially they did not trust the school/teachers to protect their child. So while the school may have been struggling to address the issue of bullying, this was not how it was perceived by parents who felt they were being kept at a distance. This is problematic because evidence has suggested that teachers sometimes avoid interacting with parents who make complaints, and these parents can be perceived by schools as bad parents, when in fact the parents are endeavouring to be the opposite of this. Research has also shown that parents’ perceptions of invitations from children and teachers to be involved in school are the most consistent predictors of their involvement (Walker et al. 2010). Collectively these issues highlight the risk of an impasse emerging in parent–teacher collaboration in school bullying situations.

The data revealed some differences in the concerns of parents depending on whether their child was in primary or secondary school. Secondary school parents felt that they were more external to the school in comparison to experiences at primary school. Parents of secondary school children also reflected on the difficulties in protecting their child as they progressed through adolescence and the expectation that they would have to support their child to cope with these sorts of problems more independently. However, despite these age specific considerations, there were significant commonalities in parents’ experiences including their frustrations with schools/teachers when the problem was not tackled, their drive to protect their child, self-scrutiny about their role as a parent, and feeling powerless to help their child.

The sense of helplessness felt by parents in this study was exacerbated when teachers failed to keep in contact with them and/or referred them to an anti-bullying policy document without appearing to follow the guidelines it contained. Previous research has also demonstrated that parents often feel that school staff are unable or unwilling to enforce their policies (Brown et al. 2013; Harcourt et al. 2015). However, it should be noted that in this study three parents talked about positive experiences with their child’s new school/teachers. The key difference in their experiences was the level of communication between parents and teachers. Good communication meant they had trust in their child’s teachers to address the problem.

Previous research has shown that schools do not always take parents’ complaints seriously, especially if their complaint pertains to verbal or indirect forms of bullying (Naylor et al. 2006; Yoon et al. 2016). Schools can also be intimidating for parents, appearing not to value their input and maintaining control during parent-teacher interactions (MacLure and Walker 2000). Researchers have suggested that parent-teacher relationships are often characterised by tension, and in particular this is because of the different perspectives that teachers and parents hold about their own role, and each other’s role. In this study, the parents saw their main role as protecting their child, and they expected teachers to do the same. Frustration, anger and upset were directed towards teachers/schools that appeared not to be protecting their child, because the bullying persisted. As shown in Brown et al. (2013) study, if parents believed that the school was not tackling the problem, they would intervene and take measures to protect their child including moving their child to another school.

Roffey (2004) also found that parents believed the parental role was to protect their child, and they expected teachers to do this on their behalf. Whereas teachers highlighted that it was their responsibility to balance the needs of one child against the needs of other children and deliver good quality education (Ribbens McCarthy and Kirkpatrick 2005). Additionally, teachers viewed ‘good’ parents as those who support teachers and do not question their decisions, and ‘bad’ parents as those who do the opposite to this (Graue and Brown 2003). However, from the parental perspective, a good parent did challenge teachers if they thought that teachers were not acting in their child’s best interests (Roffey 2004). From this it becomes understandable how the tricky parent-teacher relationships that parents in this study talked about, might have emerged.

The concept of the good parent, and what constitutes being a good parent was an important theme in this research. For the parents in this study, protecting their child was viewed as their main role as a parent; it was instinctual and fundamental to the parental role. In the focus groups/interviews, a number of processes in relation to this concept could be noted: (a) the parents (in general) viewed themselves as good parents and could provide examples to support these self-appraisals; (b) according to the parents, a good parent protected their child, instilled good values (thus, preventing bad behaviour) and prepared their child for adulthood by granting increasing autonomy; (c) when their child was bullied, the parent needed to protect their child however; (d) protecting their child was often not possible for these parents, especially if they perceived institutional factors were hindering their efforts; (e) this created dissonance because the parent recognised themselves to be a good parent, yet their inability to protect their child meant they sometimes doubted themselves and finally; (f) this led to a number of responses, including frustration towards the school, challenging the school’s handling of the problem, and self-blaming/guilt for their child’s experiences.

Research has suggested that parents, professionals and society position parents in one of two polarised groups: ‘good’ and ‘bad’ (McCormick 2010; Pedersen 2016; Phoenix and Woollett 1991). Evidence has also shown the impact of parents’ self-scrutiny, including guilt, anxiety, and a lowered self efficacy in mothers who put pressure on themselves to be the perfect parent (Henderson et al. 2016). Thus, it is perhaps unsurprising that parents who deemed themselves to have not protected their child came to doubt themselves as being good parents. Notions about what it means to act as a good parent in a school bullying situation have emerged in other studies and signalled the importance of examining parents’ experiences within the context of their perceptions of the parental role. For example, Hein (2014) found that parents engage in a process of looking inwards at themselves to determine how they might have contributed to their child’s victimisation experiences. In this situation parents can doubt their actions and feel powerless to help their child. Parents in the research by Harcourt et al. (2015) also expressed a sense of failure and guilt at not being able to protect their child.

### Limitations

According to Treharne and Riggs (2013, p. 63) analytical generalisation is “the process of generalising from some data to an extant theory rather than generalising from some data to the population, as is attempted in statistical generalisation”. The themes that emerged in this study parallel the findings of other research in this area and the broader theory of the perceived role of a good parent. In this way, the findings from this research appear to resonate with the experiences of other parents. Even so, it is important to note that the experiences of parents who participated in the focus groups/interviews may not be representative of the experiences of all parents whose children are being bullied. In cases where teachers assisted with recruitment, some parents may not have been considered, for example those who had tricky relationships with the school or those who found interactions with teachers intimidating. There will also be bias in the parents who volunteered. This is because the participants shared a common endeavour to protect their child, and this may have contributed to their decision to participate in the study. Moreover, those who had an especially negative experience may have been more motivated to participate in the research to flag up the problems they experienced (especially problems in relation to the school). Accordingly, this sample is unlikely to be representative of all parents of bullied children. Similarly, the experiences they had when working with their child’s school only showed what they encountered and are not necessarily the norm.

It was disappointing that no fathers/male carers participated in the research despite the invitation to participate being open to both parents. The issue has been encountered in other studies targeted at parents (for example, Holt et al. 2009 and Sawyer et al. 2011). Research with fathers/male carers is essential because they are likely to play a crucial role in children’s experiences, especially in their sons’ experiences. Studies are also needed to explore the extent that fathers’ responses to children’s peer victimisation are different to mothers’ responses. Future research should also examine the experiences of parents whose children engage in bullying behaviours. The parents in this study were only asked about their child’s experiences of victimisation and were not asked to consider whether their child could have bullied others. However, learning about the experiences of parents of children in other roles such as ‘bully’ and ‘bully-victim’ should not be overlooked in future studies.

### Implications for Research

It is important to acknowledge that while an overall negative account of interactions with schools/teachers is given in this paper, there were some examples of positive experiences. It is also essential to recognise the competing demands on teachers’ time and conflicting opinions about what should be their focus including tackling behaviour problems, completing education and administration tasks, and supporting students who have individual personal problems (Brown et al. 2002; Mulholland et al. 2013). Teachers have reported pressure from parents to achieve good results, say they are held entirely accountable for pupils’ examination grades, and are often met with disparaging parental opinions about the teaching profession (Brown et al. 2002; Brown and Manktelow 2016). This paper is not intended to add to the criticism aimed at teachers, but instead highlight the parental perspective and give insight into their responses. For instance, parents who regularly intervene in teachers’ handling of school bullying incidents are likely to be doing so out of concern to protect their child, rather than because they doubt the teachers’ expertise. Indeed, it is acknowledged that many teachers act strenuously to tackle bullying in their schools. This research emphasises the importance of schools communicating this to parents to prevent them from suspecting indifference in the teachers. Other studies have highlighted the perils of teachers not relaying their actions to parents. In this situation, parents will use their child’s reports of their experiences (for example, continued victimisation) as an indication of what the school has (or has not) done to tackle the problem (Hein 2014; Rigby 2013). These issues illustrate the need for research to explore school bullying from the teacher’s perspective, including the difficulties they may encounter when working with parents.

This was an exploratory study and provides direction for areas of future research in this field. Studies are needed to explore how parents’ experiences are influenced by the type of bullying that their child is subjected to. It should also be noted that previous research has examined a number of youth characteristics in relation to the prevalence of peer victimisation (Hong and Espelage 2012). This includes the experiences of gay, lesbian, bisexual and transgendered youth (e.g. Almeida et al. 2009; Swearer et al. 2008), disabled young people (e.g. Bourke and Burgman 2010), and the association between race/ethnicity and peer victimisation (e.g. Graham 2006; Vervoort et al. 2010). The focus of this study was to explore parents’ experiences, and while youth characteristics were occasionally mentioned by the parents, for instance one parent referred to her child’s ethnicity, future research should explore these areas in relation to parental experiences.

The findings of this study showed how parents can struggle to view themselves as good parents, especially when they feel frustration towards perceived institutional factors. Schools will encounter numerous challenges addressing the issue of bullying, but this is not how it is perceived by the parents who feel they are being excluded from the process. This can result in parents being viewed by the school as bad parents when in fact their intentions are to be good parents. Therefore, schools need to enter into an alliance with parents if they are to develop a truly effective strategy. This is likely to be more achievable when each party has a better understanding of the other’s perspective. A positive starting point would be for teachers to understand and acknowledge the parents’ principal role as the ‘protector’ and ‘defender’ of their child (Roffey 2004). Similarly, parents do not always see the work that is being done by teachers to tackle the problem and can incorrectly deduce that no action has been taken. Thus, it is important that anti-bullying policies include clear information about how parents can contact the school, and when and how teachers will communicate with them. Crucially, both parties need to endeavour to meet their responsibilities stated in the policy. This is likely to be more feasible for parents if they have had an opportunity to contribute to the content of the anti-bullying policy including what they see as their responsibilities, their preferred method(s) of communication and advice for how to help their child. This research reminds us that while it can take time for schools to establish what happened and respond accordingly, parents’ accounts and viewpoints need to be heard and taken seriously (Rigby 2008).
